# The Bath Case and Its Lessons

**Published:** 1917-09-15

**Authors:** 


### Adequate Payment for Whole-Time Services.

### The Bath Case and its Lessons.

The Bath Education Committee, according to the report of its proceedings published in the *Bath Chronicle*, recently sought a successor to Dr. Mary Morris, who for nine years had carried out the work of school medical inspection and who had resigned her appointment. The sub-committee wished to advertise for a temporary whole-time medical officer at a salary of £300 a year, but, although such an advertisement was accepted by "two what might be called local government journals," it was not accepted by any medical journal. The sub-committee interviewed local representatives of the British Medical Association, and it was decided to advertise the post at £500.

It is satisfactory to note the effect of this steady pressure which has been put upon public bodies in the endeavour to prevent the payment of inadequate salaries to their medical officers. There is no combination of medical men of the nature of a "trades union," and the financial interests of the profession as a whole have suffered as a consequence of the dignity, the isolation and the independence of its members. The fact is that public bodies have only recently come to realise that a medical officer must, like any other employee, be paid a salary in some degree commensurate with the cost of his education and with his position in life. Under the lead and influence of the voluntarily supported hospital system doctors have often, perhaps too often, held appointments which were honorary or for which only a small honorarium was payable. With a shorter hospital curriculum newly qualified men used to accept appointments for the sake of practice, especially if the work allowed time for reading or for preparation for further examinations. These and similar influences are passing, and such cohesion as now exists between medical men to enforce the payment of adequate salaries to the holders of public appointments may be regarded as a portent of good omen and as a sign of the times in which we live.

